# Innovative Population and Intervention Research for LGBTQ+ Older Adults With Dementia in a COVID World

**DOI:** 10.1093/geroni/igab046.425

**Published:** 2021-12-17

**Authors:** Charles Emlet, Karen Fredriksen Goldsen

## Abstract

This past year, the lives of vulnerable older adults, including those within the older LGBTQ+ community have been disrupted dramatically, as has the research agendas designed to improve their lives. Older people, including LGBTQ+ older adults with dementia, have been placed at increased risk for social isolation and mental health issues during COVID, making viable interventions even more crucial. Additionally, how research is conducted within these communities needed to be adjusted in order to preserve viability. This symposium draws upon data from the National Health Aging and Sexuality/Gender study, the first longitudinal study of LGBTQ+ older adults in the United States, as well as data from Aging with Pride: IDEA (Innovations in Dementia Empowerment and Action), the first randomized controlled trial (RCT) designed to improve quality of life of LGBTQ+ adults living with dementia and their care partners. (1) Kim and Fredriksen Goldsen examine modifiable behavioral and social factors that can improve quality of life among LGBTQ+ older adults with cognitive impairment. (2) Fredriksen Goldsen, Teri, Emlet and colleagues present initial efficacy findings from the IDEA study and how the intervention needed to be altered to be viable in a COVID world. (3) The importance of Motivational Interviewing (MI) as part of a LGBTQ+ sensitive intervention designed for LGBTQ+ older adults with dementia and their care partners is discussed by Petros, Fredriksen Goldsen and Teri. As COVID continues to impact vulnerable populations as well as research and service delivery, identifying new and innovative strategies will become increasingly important.

